# Trust, risk perception, and intention to use autonomous vehicles: an interdisciplinary bibliometric review

**DOI:** 10.1007/s00146-024-01895-2

**Published:** 2024-03-25

**Authors:** Mohammad Naiseh, Jediah Clark, Tugra Akarsu, Yaniv Hanoch, Mario Brito, Mike Wald, Thomas Webster, Paurav Shukla

**Affiliations:** 1 Department of Computing and Informatics, University of Bournemouth, Bournemouth, UK; 2 Faculty of Engineering and Physical Sciences, University of Southampton, Southampton, UK; 3 Southampton Business School, University of Southampton, Southampton, UK; 4 Centre for Business in Society, Coventry University, Coventry, UK; 5 Web and Internet Science Group, Faculty of Engineering and Physical Sciences, University of Southampton, Southampton, UK; 6 Connected Places Catapult, London, UK

**Keywords:** Autonomous vehicles, Trust, Risk, Bibliometric analysis

## Abstract

Autonomous vehicles (AV) offer promising benefits to society in terms of safety, environmental impact and increased mobility. However, acute challenges persist with any novel technology, inlcuding the perceived risks and trust underlying public acceptance. While research examining the current state of AV public perceptions and future challenges related to both societal and individual barriers to trust and risk perceptions is emerging, it is highly fragmented across disciplines. To address this research gap, by using the Web of Science database, our study undertakes a bibliometric and performance analysis to identify the conceptual and intellectual structures of trust and risk narratives within the AV research field by investigating engineering, social sciences, marketing, and business and infrastructure domains to offer an interdisciplinary approach. Our analysis provides an overview of the key research area across the search categories of ‘trust’ and ‘risk’. Our results show three main clusters with regard to trust and risk, namely, behavioural aspects of AV interaction; uptake and acceptance; and modelling human–automation interaction. The synthesis of the literature allows a better understanding of the public perception of AV and its historical conception and development. It further offers a robust model of public perception in AV, outlining the key themes found in the literature and, in turn, offers critical directions for future research.

## Introduction

The automotive industry and driving itself are undergoing a revolution, with the development of autonomous vehicles (AVs). Although the AVs are still in the experimental phase, they are expected to become widely available and affordable in the near future. Transport planners and urban designers anticipate that autonomous vehicles will considerably alter transportation systems (Litman 2020). Indeed, autonomous vehicles are becoming a frequent topic in mainstream media, with some reports highlighting that “driverless cars will change our world” (Cusack 2021), while others raise concerns about their safety, including incidents of collisions (Bateman 2021). Despite the significant technological advancements made in this domain, AVs still face numerous societal challenges (Cugurullo and Acheampong 2023; Lundgren 2021). Some of the major societal challenges that remain unresolved include drivers’ acceptance of autonomous vehicles as well as legal, and ethical issues (Sprenger 2022; Gaio and Cugurullo 2022). Additionally, the acceptance of novel technologies often hinges on the public’s perceived risk and trust (Zhang et al. 2019a, b). To explore the issues of risk perception, trust, and other related concerns that are crucial for the success of the adoption of novel technologies, this review aims to provide readers with state-of-art insights about our present knowledge as well as future directions.

Defining human understandable automation levels in vehicles remains as a challenge. One study, for example, indicates that the public is still confused about the capabilities of vehicle automation, because they cannot understand their role in the vehicle (Abraham et al. 2017). The term ‘*autonomous vehicle*’ (AV) is a broad definition for any road vehicle capable of managing and conducting driving tasks (EU 2019). AVs can be further defined as being *partially automated* (longitudinal or longitudinal control is automated, but only under certain conditions) or *fully automated* (a vehicle requiring no human input outside of strategic decisions such as destination management) (SAE 2019). These systems are collectively known as ‘automated driving systems’ (Koopman et al. 2021; NHTSA 2021).

Many vehicles today are equipped with advanced driver assistance systems (ADAS), which differ from automated driving systems (ADS) in critical ways. These systems require continuous human monitoring and incorporate features such as automatic emergency braking and lane centring assist (NHTSA 2021). Additionally, many vehicles may be equipped with advanced safety features such as forward-collision warnings, lane departure alerts, and blind spot detectors (NHTSA 2021). In this regard, the SAE J3016 standard categorises six levels of AV automation, ranging from Level 0 (no automation) to Level 5 (full automation without a steering wheel).

This AV automation classification starts with Level 0, where there is no automation and the human driver has full control over the car. Level 1 introduces single-driver assistance features that supports the human driver in steering or acceleration/deceleration, but the driver remains in charge of car operation and can take control at any time. In Level 2, AVs manage both steering and acceleration/deceleration, yet the driver must actively supervise the car at all times. Level 3 brings advanced automation capabilities, which enables the car to respond to changes in the driving environment. However, similar to Levels 1 and 2, drivers in Level 3 must be alert and able to take control at any time. Level 4 AVs are capable of complete journey on the highways and in city traffic independently. While Level 4 AVs do not require any human interaction, human drivers can take back control of the car under certain conditions, such as extreme weather. Finally, Level 5 represents the pinnacle of automation: full automation where humans have no control over the car under any conditions and Level 5 vehicles do not feature a steering wheel.

Substantial research and resources are being invested in the development and success of semi-autonomous (Level 3–4) and fully autonomous vehicles (Level 5) (Fagnant and Kockelman 2015). However, an increased level of automation does not automatically translate to improved system performance or user acceptance. Therefore, understanding public trust and risk perceptions becomes crucial for the successful adoption and acceptance of AVs.

Research suggests that the implementation of advanced safety features is expected to reduce crash rates in the USA (IIHS 2020). The study conducted by the Insurance Institute for Highway Safety (IIHS 2020) highlights a 50% decrease in front-to-rear collisions, a 14% drop in lane-change collisions, and an 11% reduction in single-vehicle, sideswipe, and head-on collisions. Similar to advanced safety features, ADS and ADAS are expected to enhance road safety and provide our societies with a more inclusive future. There are ongoing debates in the research community regarding the potential of AVs to assist vulnerable groups such as the elderly and those with disabilities, where different scenarios are considered (Harper et al. 2016). However, the growing complexity and inherent uncertainties associated with automated driving systems—as is common with the introduction of new technology (Bagloee et al. 2016)—may lead to a variety of trust issues. These issues might impede adoption and increase the potential public scepticism or opposition (Liljamo et al. 2018).

Global efforts are underway to develop AV technologies (SMMT 2019; Intel 2016) and to establish public policies for the safe and efficient rollout of partial and full automation on public roadways. The pace at which AV technology can be implemented is highly dependent on how road users perceive and engage with AVs. Key questions are whether users will trust AVs to act in a way that they deem safe for themselves and others, whether they believe their usage data will be handled ethically and not used against them, and if they will choose to use AVs in certain situations over others based on perceived risks, such as in complex versus simple driving conditions. These questions are critical in the research and development of AVs and demand further scrutiny and policy direction in this rapidly evolving field. Addressing these questions is vital for governments, policymakers, manufacturers, insurers, drivers, and other road users, as it will provide valuable insights into the development of trustworthy AVs and inform future AVs designs and policy recommendations.

Trust is a multifaceted and intricate concept, therefore challenging to define succinctly. In his seminal work, Goffman (1963) emphasises the central role of trust in the process of social acceptance. The early definitions of trust that emerged in psychology and sociology (Rotter 1967) highlighted the positive consequences of trust to individuals and society as a whole, as well as demonstrated the key role that trust plays in establishing meaningful and cooperative relationships. This view was further expanded through the lens of uncertainty, vulnerability, and dependence, wherein Moorman et al. (1993) assert that trust is a willingness to rely on an exchange partner in whom one has confidence. Such conceptualisations highlight the sense of uncertainty and vulnerability in one party, which requires them to willingly place a degree of dependence and confidence in another party.

In various bodies of literature, trust is identified as a crucial element in decisions based on risk/benefit analysis. For instance, within the health literature, vaccine acceptance is shown to depend on public trust and confidence (e.g. Larson et al. 2018). Similarly, trust is considered as a fundamental factor for the adoption of emerging technologies, such as blockchain (e.g. Shin 2019). However, defining trust, especially in the consumer contexts, remains elusive. A systematic review concluded that only few studies explicitly define the nature of consumer trust (Wang et al. 2014). Similarly, a comprehensive review of organisational trust literature, encompassing 171 papers spanning over 48 years of research identified 129 different definitions of trust that had been operationalised into 38 different dimensions (McEvily and Tortoriello 2011). These reviews clearly highlight the difficulties associated with the ongoing challenges in the conceptualisation and understanding of trust.

Operationalising trust in also presents significant challenges, particularly due to difficulties in directly measuring it as a construct (French et al. 2018). Many researchers opt for subjective measures of trust post-study, which may overlook critical real-time trust factors that may be more relevant (Desai et al. 2013). Trust is also conceptualised as an ‘attitude’ that manifests in the physical act of reliance (i.e. using the system), considered as ‘behaviour’ (Choi and Ji 2015). However, reliance does not always directly correlate with the level of trust, as highlighted by Lee and See (2004)’s seminal model for trust in automation. A high level of trust can lead to inappropriate reliance, such as activating automation in unsafe conditions, where the system cannot perform safely resulting in a collision. Conversely, a low level of trust, compared to system reliability, can lead to an underutilisation of the automated system, resulting in a loss of benefits.

At a societal level, various factors, such as brand reputation, media influence, risk perception, perceived usefulness, public acceptance, and individual experiences will inevitably contribute towards the complex interactions leading to the uptake (i.e. reliance) of the AV technology (Choi and Ji 2015; Feldhütter et al. 2016; Hulse et al. 2018; Gold et al. 2015; Lee and See 2004; Walker et al. 2020). The degree to which these factors are effectively addressed and the extent to which trust is well calibrated will largely depend on public policy, effective marketing strategies and building relationships with road users over time (Straub and Schaefer 2019; Yuen et al. 2020; Zhang et al. 2020).

Several researchers have examined trust towards AVs and identified important parameters (e.g. Waung et al. 2021; Zhang et al. 2019b). For instance, the role of socio-demographics such as age (Dikmen and Burns 2017), socio-psychographics such as experience, perceived ease of use (Xu et al. 2018), environmental factors such as weather and road conditions (Ha et al. 2020) and AV-related issues such as unexpected behaviour and anthropomorphism having substantial influence on trust (Niu et al. 2018; Ruijten et al. 2018; Lee and Lee 2022). Moreover, trust has been identified as a key precursor to a variety of attitudinal and behavioural factors such as frequency of use, self-rated knowledge about these systems, and ease of learning (Dikmen and Burns 2017; Choi and Ji 2015; French et al. 2018).

Similar to trust, the field of risk perceptions is expansive, interdisciplinary, and embraces diverse viewpoints and methodologies. Risk is a concept that has been extensively explored in both physical and social sciences, recognised as a pivotal factor influencing public perceptions (Breakwell 2014). In physical sciences, risk is often approached through the lens of the probabilistic occurrence of an adverse event. In contract, in social sciences it is argued that the public do not engage in exact probabilities but rather rely on intuitive risk judgements (Slovic 2000). Studies have shown that public perceptions of risks are influenced by a variety of factors including, familiarity, control, catastrophic potential, equity, and level of knowledge (e.g. Huang et al. 2013; Mayeda and Boyd 2020). These factors collectively influence the relationship between perceived risk, perceived benefit, and the acceptance of risk. Consequently, perceived risk is found to influence emotional responses such as the levels of concern, worry, anger, anxiety, fear, hostility, and outrage, resulting in a significant change in attitudes and behaviour of people (for a review see, Ferrer and Klein 2015).

In the context of AVs, research into risk perceptions uncovers important implications. Xu and Fan (2019) argue that Chinese consumers anticipate lower risks with AVs, and thus expecting lower insurance premiums for such vehicles, while emphasising the importance of familiarity and personal information. Similarly, Chikaraishi et al. (2020) reveal that unfamiliarity and other emotions such as dread play an important role in shaping Japanese consumers’ public risk perceptions of AVs. In their meta-analysis, Nishihori et al. (2020) found that factors such as gender, population density in the area, and familiarity can reduce risk perception related to AVs. Brell et al. (2019), also highlight the role of experience in mitigating risk perceptions among German drivers, although they stated that experience does not alter perceptions regarding data handing perceived risk associated with AVs.

The literature presents several attempts aimed at examining autonomous vehicles from diverse perspectives. Gandia et al. (2019) conducted a comprehensive review of existing literature on autonomous vehicles, encompassing their characteristics and revolutionary aspects. Di Ciaccio and Troisi (2021) took a different approach, combining bibliometric and social network analyses to investigate the utilisation of autonomous underwater vehicles (hereafter AUVs) in environmental monitoring operations and to identify potential future areas of applications for AUVs. Tal and Gordon (2020) conducted a bibliometric analysis to address the question whether leadership represents a developing area within the field of autonomous research. Conversely, Silva et al. (2020), delved into the research domain of autonomous vehicles and terrestrial mobility to unravel the primary trends and studies pertaining to autonomous vehicles.

As of present, there is no comprehensive benchmark assessing the state of public perceptions and future challenges associated with the adoption of autonomous vehicles, relating to both societal and individual barriers to uptake, particularly concerning the vital aspects of trust and risk perceptions—factors that are crucial for the success of any emerging technology. Our study, therefore, distinguishes itself in several ways from the current bibliometric analysis in the literature (a comparison is outlined in Table 1). Firstly, by examining the extensive, yet fragmented and cross-disciplinary research on AVs, our aim is to synthesise the existing body of work and provide a clear structure for various key players who seek to understand trust and risk perceptions related to autonomous vehicles. Secondly, we aim to establish a comprehensive benchmark that can support the international development and deployment of AV technology in a safe and responsible manner.

**Table 1:** Comparison of previous studies on AV and our study

Article	Aims of the study	The focus of the study	Time period	Methodology
Gandia et al. (2019)	To present a bibliometric review of autonomous vehicles	To identify the main characteristics of AV, main themes, and evolution	1945–2018	Descriptive analysis, dual map overlay analysis (to identify main disciplines), categories review
Di Ciaccio and Troisi (2021)	To explore autonomous underwater vehicles (hereafter AUV) and the literature	To assess the current and potential working areas of AUV, to investigate the existing network of scientific development	1995–2019	Co-authorship, co-occurrence, and citation analysis
Tal and Gordon (2020)	To understand the leadership as the research area in AV	To answer ‘is leadership as a research area developing into an autonomous research field?’	1960–2020	Descriptive analysis
*Our study*	To understand the trust and risk perception within autonomous vehicles	To highlight the key considerations for trusting an AV both on a societal and individual level, and identify research opportunities for future research in trust, public acceptance, and risk perception across all levels of automated functionality	1980–2020	Descriptive analysis, co-citation analysis

A key focus of our study is the exploration of societal and individual challenges at various levels of automation. We posit that understanding trust in the context of specific automation levels, such as such as partial automation, may not readily translate to all other levels of automation. To address this gap, we conducted a bibliometric analysis, to highlight the key considerations for trusting an AV both on a societal and individual levels, and identify research opportunities for future research in trust, public acceptance, and risk perception across all levels of automated functionality. In doing so, the public and stakeholders will benefit from the development of trustworthy automated technology that serves the priorities of the public about ethics, health, and day-to-day lifestyle. Table 1 compares earlier reviews of the autonomous vehicles research and our contribution on several dimensions.

In our study, we address existing research gap through an analysis that employs bibliometric and performance techniques. The main aim of this investigation is to identify the conceptual and intellectual frameworks that underpin trust and risk narratives in the field of AV research. To achieve this, our study spans various disciplines, including engineering, social sciences, marketing, business and infrastructure. This interdisciplinary research enables a more holistic understanding of the trust and risk narratives associated with AVs.

Our methodology includes a performance based on citation and publication data, allowing us to evaluate the scientific productivity and identify the key scientific actors contributing to the autonomous vehicle research field. Given the complex and multifaceted nature of AV research, as well as the involvement of various stakeholders (e.g. governments, policymakers, manufacturers, insurers, drivers, and other road users in the development of the area), our study aims to capture the core themes within the trust and risk narratives pertaining to the AV research domain. By doing so, our objective is to expand the existing body of knowledge and to provide a roadmap that can inform decision-makers and scholars studying this important field.

## Method

### Search strategy and selection of database

For our study, we selected Web of Science (WoS) as a bibliographic database, recognised for its comprehensive coverage for multiple disciplines^1^ (e.g. Maisonobe 2022; Pranckute 2021). To ensure a comprehensive exploration of trust and risk narratives within the field of autonomous vehicles (AV), from an interdisciplinary perspective, we consulted 21 domain experts.^2^. These experts provided valuable insights and helped us to identify primary and synonymous keywords for literature searches in this specific domain. By using Boolean operators ‘AND’ or ‘OR’, we aimed to achieve inclusivity in our research. The keywords that are used to retrieve the studies are as follows: Trust AND (Autonomous vehicle OR Automated vehicle), Risk AND (Autonomous vehicle OR Automated vehicle). Following the expert guidance and the selected database, we identified five major research disciplines for our focus: (1) engineering, (2) social sciences, (3) marketing, (4) business, and (5) infrastructure domains.

During the search process, we scrutinised four key fields, namely title, abstract, keyword, and reference identifiers as well as manuscripts (where necessary) to determine their relevance to the domain and the appropriate utilisation of the selected keywords). Our inclusion criteria were stringent, in that only papers published in English within the timeframe of 1980–2020 were to be considered Consequently, we included only those papers that fulfilled all our predefined criteria in our study’s database. We excluded indirect research materials such as editorials or book reviews from the analysis, whereas conference proceedings and reports were included in the analysis due to their importance in the engineering field (Kochetkov et al. 2021). This approach resulted in an initial set of 936 documents spanning over four decades. To ensure research validity, two researchers independently applied the selection and inclusion criteria (Zupic and Cater 2015).

### Analysis

Bibliometrics is often characterised as a qualitative-driven quantitative approach for analysing and assessing a research domain (Verma and Yadav 2021; Chabowski and Mena 2017; Chabowski and Samiee 2020). This methodology encompasses various analytical techniques developed for different types of assessments within a research domain. The assessments generally fall into two main categories: performance and science mapping analysis (Moral-Muñoz et al. 2020). Performance mapping aims to illustrate the scientific actors and production growth in research output, while science mapping aims to present the knowledge structure of a research domain through co-citation analysis (Donthu et al. 2021).

In our study, we used R programming language to conduct performance analysis, encompassing elements such as the most relevant sources and the annual growth rate. In terms of science mapping, an approach that explores intellectual interactions among research constituents (Baker et al. 2021), multiple methodologies are recommended such as co-word analysis or co-authorship analysis (Donthu et al. 2021). We used co-citation analysis. This approach not only reveals the intellectual structure of the field, but also facilitates the identification of the knowledge structure through an examination of the most frequently cited articles (Hjørland 2013). Furthermore, it allowed us to understand how the AV domain intersected with other research areas. By employing co-citation analysis, we identified the knowledge structure through the most cited articles as well as understood how the AV domain has been incorporated with other study streams.

To maintain transparency and ensure a systematic and reproducible investigation (Verma and Yadav 2021), we followed PRISMA (Preferred Reporting Items for Systematic Reviews and Meta-Analysis Statements) guidelines throughout the stages of keyword identification, data screening, and inclusion (Page et al. 2021). Once the database was collected through Web of Science (WoS), we coded the data for consistency and transferred it into Bibexcel for citation analysis (Persson et al. 2009). The process involved using citation frequency as a metric to rank papers that significantly contribute to defining the knowledge structure of trust and risk narratives within the autonomous (Samiee et al. 2015; Chabowski and Samiee 2020; Pasadeos and Renfro 1989). The most cited papers on trust and risk narratives within the AV field can be found in Table 2.

**Table 2:** The most highly cited documents on trust and risk perception in the AV field

Authors	Article	Journal/publisher	Domain (trust/risk)	Key concerns
Fagnant and Kockelman (2015)	Preparing a nation for autonomous vehicles: opportunities, barriers and policy recommendations	Transportation Research Part A-Policy and Practice	Risk and trust	Costs, liability, regulation, policy, security, and data privacy
Kyriakidis et al. (2015)	Public opinion on automated driving: Results of an international questionnaire among 5000 respondents	Transportation Research Part F-Traffic Psychology and Behaviour	Risk and trust	Acceptance, concerns, and willingness to buy
SAE (2019)	Taxonomy and Definitions for Terms Related to Driving Automation Systems for On-Road Motor Vehicles (J3016_202104)	SAE International	Risk	Taxonomy of the levels of automation
Bonnefon et al. (2016)	The social dilemma of autonomous vehicles	Science	Risk	Public preferences for AV behaviour/life preservation
Bansal et al. (2016)	Assessing public opinions of and interest in new vehicle technologies: An Austin perspective	Transportation Research Part C-Emerging Technologies	Risk and Trust	Safety, cost, performance, demographics, ridesharing, and awareness
Choi and Ji (2015)	Investigating the Importance of Trust on Adopting an Autonomous Vehicle	International Journal of Human–Computer Interaction	Risk and trust	Intention to purchase modelled with trust and perceived risk
Haboucha et al. (2017)	User preferences regarding autonomous vehicles	Transportation Research Part C-Emerging Technologies	Risk	Long-term choice decisions, interest, environmental concerns, acceptance
Kalra and Paddock (2016)	Driving to safety: How many miles of driving would it take to demonstrate autonomous vehicle reliability?	Transportation Research Part a-Policy and Practice	Risk	How much testing is required to ensure AVs are safe?
Lee and See (2004)	Trust in Automation: Designing for Appropriate Reliance	Human Factors	Risk and Trust	Trust in automation (multi-disciplinary)
Xu et al. (2018)	What drives people to accept automated vehicles? Findings from a field experiment	Transportation Research Part C-Emerging Technologies	Risk	Before and after experiencing Level 3 AVs, how does this affect acceptance?
Hulse et al. (2018)	Perceptions of autonomous vehicles: Relationships with road users, risk, gender and age	Safety Science	Risk	How road users (apart from the driver) may be affected by AVs
Payre et al. (2014)	Intention to use a fully automated car: Attitudes and a priori acceptability	Transportation Research Part F-Traffic Psychology and Behaviour	Risk and trust	Attitudes, personality traits, and acceptance of fully automated vehicles
Anderson et al. (2016)	Autonomous Vehicle Technology: A Guide for Policymakers	RAND Corporation	Risk	Liability, policy, regulation, cost, efficiency, environmental challenges, security, and inter-vehicle communication
Petit and Shladover (2015)	Potential Cyberattacks on Automated Vehicles	IEEE Transactions on Intelligent Transportation Systems	Risk	Cyberattacks on AVs
Parasuraman and Riley (1997)	Humans and Automation: Use, Misuse, Disuse, Abuse	Human Factors	Risk and trust	Trust in automation (multi-disciplinary)
Hoff and Bashir (2015)	Trust in Automation: Integrating Empirical Evidence on Factors That Influence Trust	Human Factors	Trust	Review recent empirical research on factors that influence trust in automation to present a three-layered trust model
Jian et al. (2000)	Foundations for an Empirically Determined Scale of Trust in Automated Systems	International Journal of Cognitive Ergonomics	Trust	Similarities and differences in the concepts of trust and distrust, and among the different types of trust
Lee and Moray (1992)	Trust, Control Strategies and Allocation of Function in Human Machine Systems	Ergonomics	Trust	Relationship between changes in operators’ control strategies and trust
Verberne et al. (2012)	Trust in Smart Systems: Sharing Driving Goals and Giving Information to Increase Trustworthiness and Acceptability of Smart Systems in Cars	Human Factors	Trust	Trust in smart systems
Muir and Moray (1996)	Trust in automation. Part II. Experimental studies of trust and human intervention in a process control simulation	Ergonomics	Trust	Operators’ trust in and use of the automation in a simulated supervisory process control task
Korber et al. (2018)	Introduction matters: Manipulating trust in automation and reliance in automated driving	Applied Ergonomics	Trust	Trust in automation
Waytz et al. (2014)	The mind in the machine: Anthropomorphism increases trust in an autonomous vehicle	Journal of Experimental Social Psychology	Trust	People’s willingness to trust such technology to perform competently
Davis (1989)	Perceived Usefulness, Perceived Ease of Use, and User Acceptance of Information Technology	MIS Quarterly	Trust	Development of acceptance model and measurement

After ranking the papers, we used co-citation analysis to assess the interrelationships within the research field. Based on the co-citation matrix, we could later utilise bibliometric methods, such as hierarchical cluster analysis (HCA), which is one of the most frequently used bibliometric quantitative methods for identifying the groups within a research domain based on the similarity of research (Hair et al. 1998). Through HCA, a dendrogram is generated based on the items being analysed, helping researchers to identify the ‘subgroups’ within a research domain, known as ‘clusters’ (Zupic and Cater 2015). The subgroups can be determined through a dendrogram, where the researcher should decide which item(s) will be divided into clusters, a process known as ‘cutoff’ (Janssens et al. 2008). To determine the clusters, we used Ward’s method (Reader and Watkins 2006), a connectivity-based clustering method to interpret the results. Therefore, following established bibliometric methods, we applied HCA, a quantitative method that establishes subgroups and intellectual streams of a research domain based on the similarities of each object. By using Ward’s method, a connectivity-based clustering method, we produced a dendrogram to identify the themes to have interpretable results. This iterative process led to the inclusion of 30 of the most highly cited papers in the analysis.

## Discussion

### Performance analysis

**Fig. 1:**
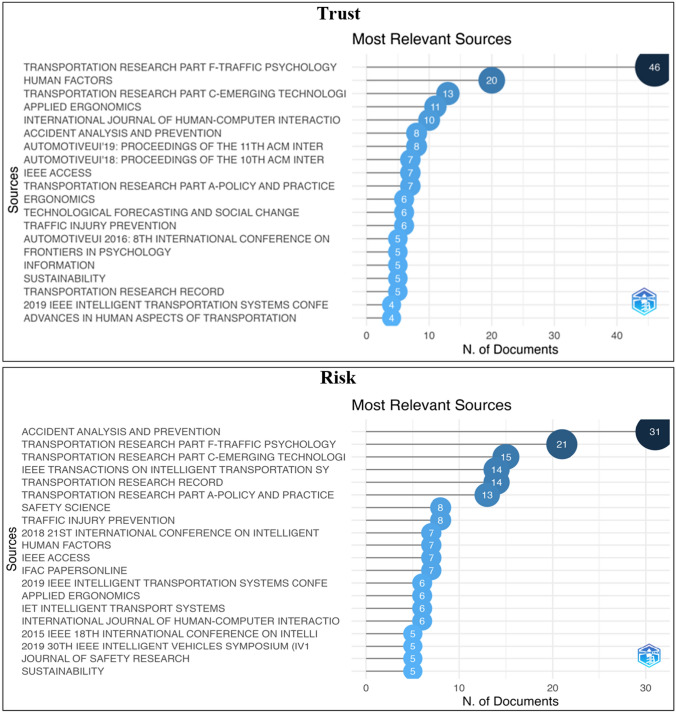
Most relevant sources for the trust and risk narratives in the AV field

**Fig. 2:**
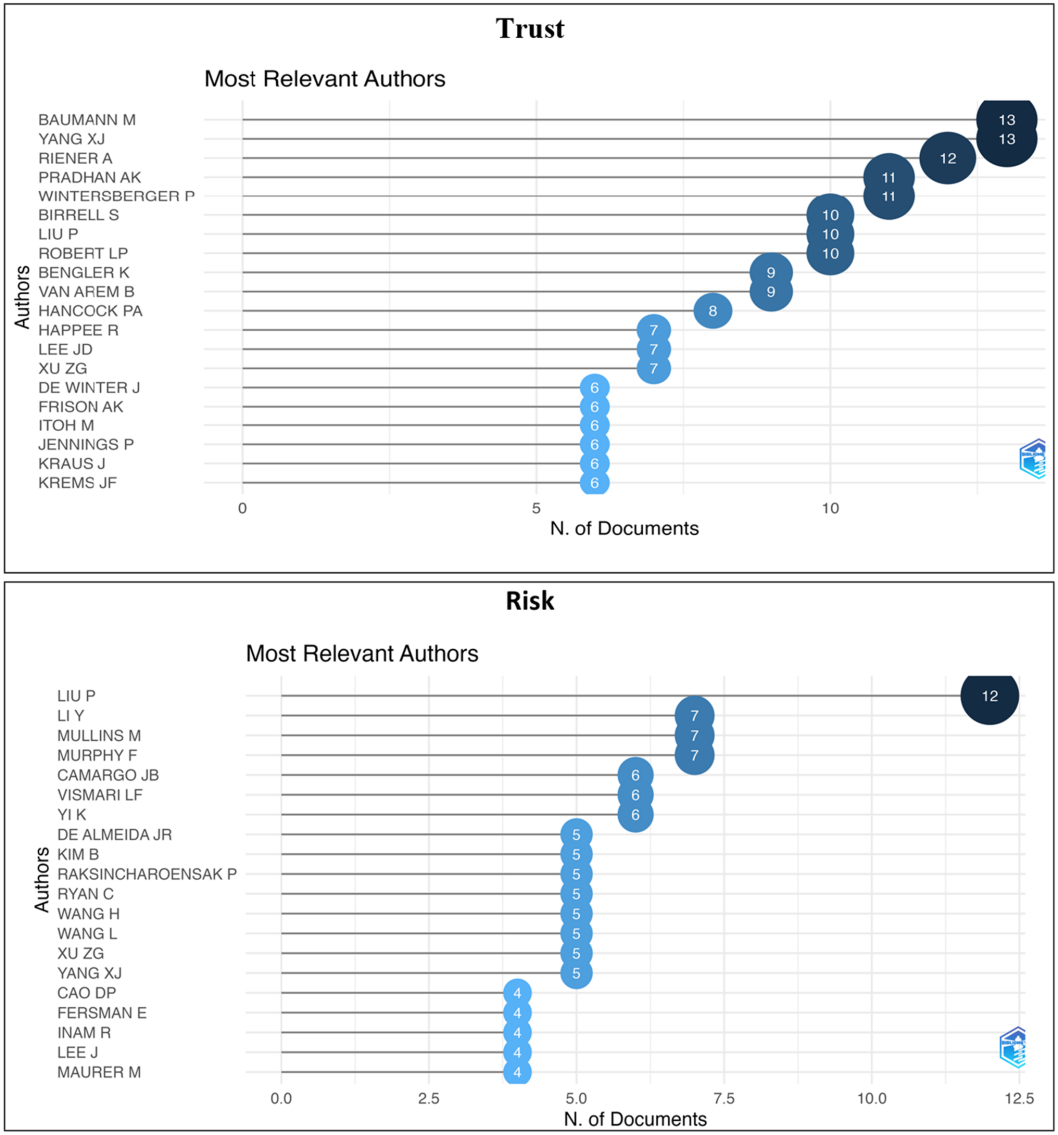
Most relevant authors for trust and risk narratives in the AV field

The publication trends in trust and risk-related research within the field of AVs in engineering, social sciences, business, and marketing domain from 1980 to 2020 are presented in Figs. 1 and 2. The results indicated that the number of papers published increased substantially over the last years. The most productive journal was found to be Transportation Research Part F-Traffic Psychology for trust, and Accident Analysis and Prevention for risk narratives, where the annual growth rates were 25.40 and 62.56%, respectively. Figure 2 presents the most relevant author and number of publications they have for trust and risk-related research within the AV domain.

**Fig. 3:**
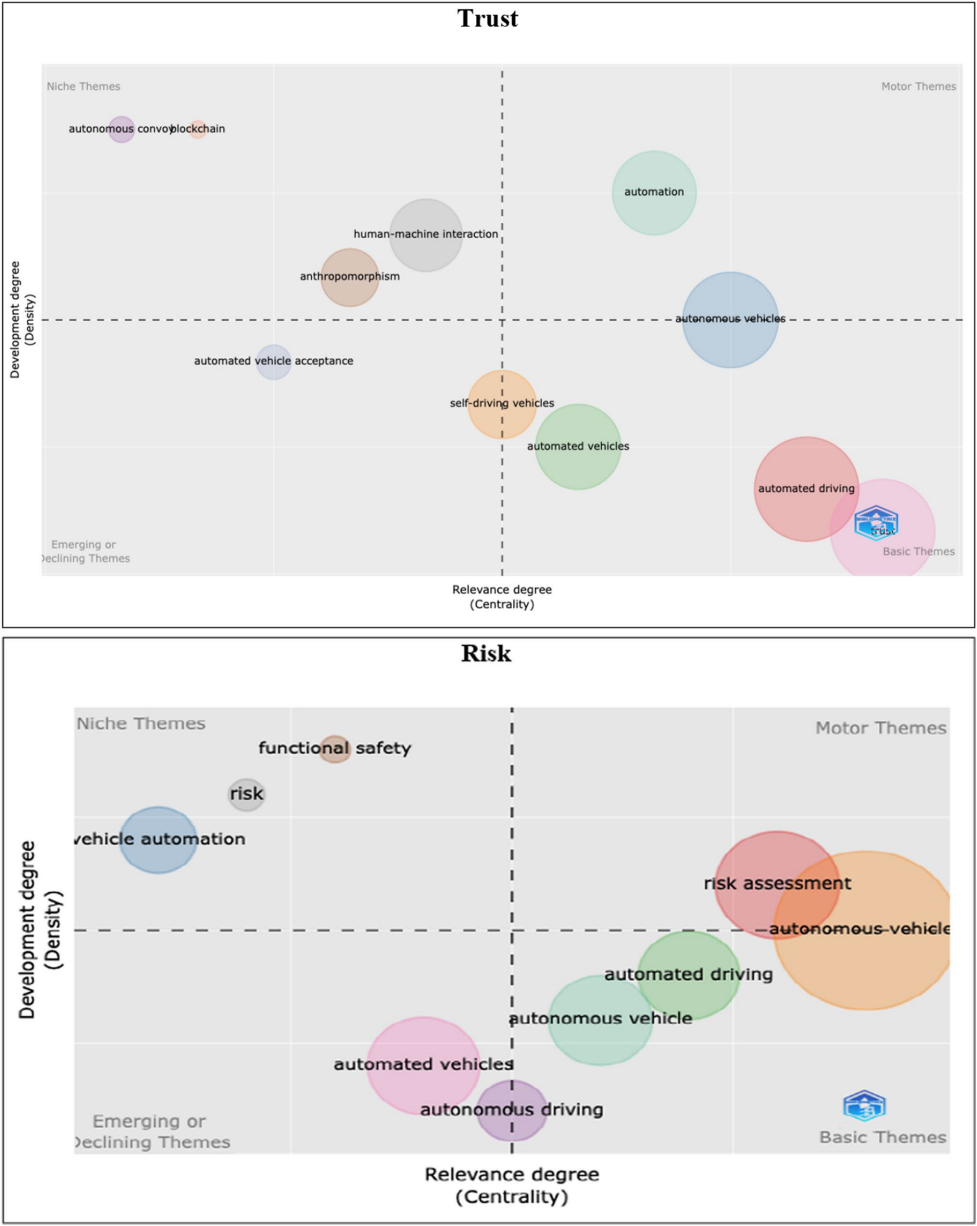
Thematic evolution of trust and risk narratives in the AV field

To understand the thematic evolution of trust and risk narratives within the AV research, we also employed a network approach, wherein the research themes are presented in two-dimensional space in four groups, based on density and centrality, namely, (1) motor themes, (2) basic themes, (3) niche themes, and (4) emergent or declining themes. Motor themes indicate the themes that are well developed and have been considered over a long period of time in field. Basic themes refer to important, but yet less developed within the field. Niche themes on the other hand are well developed, but still marginal within the field. Finally emergent or declining themes represent themes that are either not yet well developed or have received limited attention in the field (Akarsu et al. 2023; Moral-Munoz et al. 2018). As seen in Fig. 3, autonomous convoys, blockchain, human–machine interaction, and anthropomorphism are identified as niche themes. Conversely, functional safety and vehicle automation are observed as niche themes for risk. Automation and risk assessment are recognised as motor themes for trust and risk within the AV research, respectively, indicating their essential role in the research field.

### Intellectual clusters and groups

Overall, five clusters were identified across the search categories of ‘trust’ and ‘risk’. The following sections outline the definition and specification of intellectual clusters and their corresponding groups of articles.

#### Trust

**Fig. 4:**
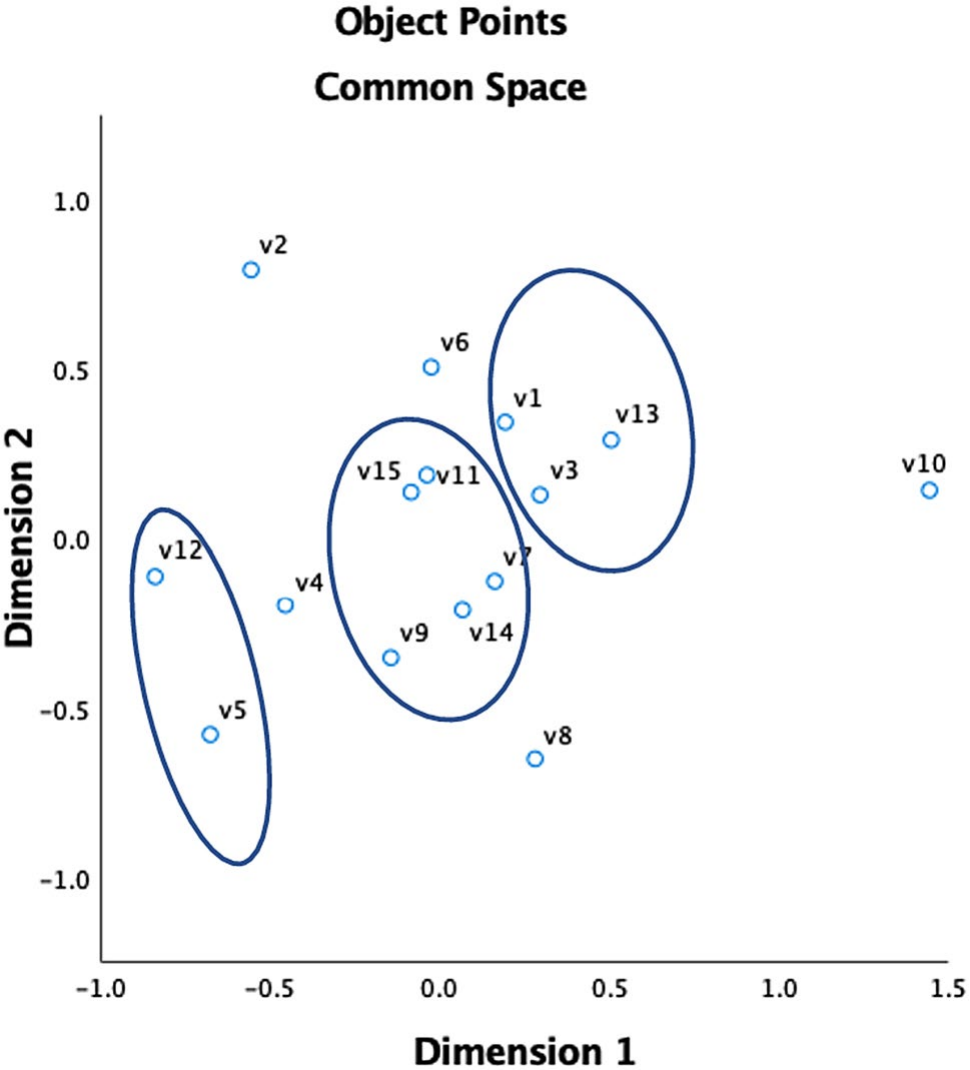
Trust: hierarchical clustering, Ward’s method. V1 = Bansal et al. (2016); V2 = Choi and Ji (2015); V3 = Davis (1989), V4 = Fagnant and Kockelman (2015); V5 = Hoff and Bashir (2015); V6 = Jian et al. (2000); V7 = Korber et al. (2018); V8 = Kyriakidis et al. (2015); V9 = Lee and Moray (1992); V10 = Lee and See (2004); V11 = Muir and Moray (1996); V12 = Parasuraman and Riley (1997); V13 = Payre et al. (2014); V14 = Verberne et al. (2012); V15 = Waytz et al. (2014)

Amongst the most cited papers during the ‘trust’ keyword search, three clusters were identified from ten of these items. These clusters represent similarities across key papers within the domain and represent three major themes within AV research. Figure 4 presents the hierarchical cluster analysis using Ward’s method. The HCA clustering led to three clusters, namely, cluster 1—behavioural aspects of AV interaction (V7, V9, V11 and V14); cluster 2—uptake and acceptance (V1, V3 and V13); and cluster 3—modelling human–automation interaction (V5 and V12) as shown in Fig. 4.

##### Behavioural aspects of AV interaction

This cluster represents the investigation into the role of the human driver in autonomous vehicle technology (see Table 3). These publications tackle issues such as how human drivers monitor and intervene with autonomous technology under certain scenarios and how these behaviours interact with perceived trust and communication with the autonomous system. These experimental paradigms typically feature simulations exploring the behavioural outcomes of the use and disuse of autonomous technology. They examine factors such as the environment (critical versus non-critical situations), pre-conceived assumptions about the system (Korber et al. 2018), pre-conceived assumptions of the system (Korber et al. 2018), and communication strategies (Verberne et al. 2012; Waytz et al. 2014).

**Table 3:** ‘Behavioural aspects of AV interaction’ cluster—summary of key research contributions

Article	Summary of work	Key themes	Level of automation
Körber et al. (2018)	Investigation of takeover performance and trust promotion—overtrust can lead to underperformance	Driving behaviour, safety, calibrating trust, takeover	2–4
Lee and Moray (1992)	Experimental paradigm exploring how humans adaptively control and intervene with automation based on trust	Safety, trust manipulation, takeover, function allocation	Unspecified
Muir and Moray (1996)	Experimental paradigm investigating how incompetence can lead to reduced trust, and the rejection of automated systems under such circumstances	Driving behaviour, safety, trust manipulation, reliability	Unspecified
Verberne et al. (2012)	Investigating the trust and acceptance of takeovers that involve the development and communication of shared goals	Driving behaviour, safety, trust manipulation, communication	2–4
Waytz et al. (2014)	The role of anthropomorphism in trust development in autonomous vehicle operation	Driving behaviour, safety, trust manipulation, communication	2–4

Both Lee and Moray (1992) and Muir and Moray (1996) support these studies by providing models and frameworks for representing the potential relationships between humans and automated systems. Lee and Moray (1992) identify the factors that affect trust over time, and how these dynamics impact performance. Muir and Moray (1996) also provide insight into how trust evolves over time, and how determinants of trust can impact whether a user will use or disuse an autonomous system. All research sources within this cluster address behavioural factors and function allocation, specifically around the safe control of systems, typically related to the optimisation of trust and task coordination.

##### Uptake and acceptance

This cluster consists of research articles predicting the uptake of autonomous vehicles and overall public acceptance (see Table 4). Investigations are primarily survey based (Bansal et al. 2016; Payre et al. 2014) and identify the key contributions towards public acceptance including the role of the environment, the impairment of the driver (Payre et al. 2014), demographics of user including location, income, gender, driving record, and pricing (Bansal et al. 2016). Davis’s (1989) technology acceptance model is the most widely adopted model for how users come to accept and perceive technology as being useful, cited over 72,700 times (Google Scholar citations at the time of writing). The article by Davis underpins the majority of technology acceptance research investigations and continues to inform the research community on how to approach individual and societal acceptance towards automated technology.

**Table 4:** ‘Uptake and acceptance’ cluster—summary of key research contributions

Article	Summary of work	Key themes	Level of automation
Bansal et al. (2016)	Large-scale survey study on willingness to pay for and openness to use autonomous vehicles of varying levels	Acceptance, usage, marketing	1–5
Davis (1989)	Original technology acceptance citation. Identifies the correlation between acceptance and usage. Provides a framework for the measurement of end-user acceptance	Acceptance, usage	Unspecified
Payre et al. (2014)	Large-scale survey on attitudes and contributors towards the acceptance of fully autonomous vehicles	Acceptance, usage, demographics	5

##### Modelling human–automation interaction

Trust is multi-faceted and can have a significant impact on many aspects of behaviour and attitudes towards technology. This cluster is concerned with the broader themes that contribute towards trust development and lays out the foundations for identifying how trust manifests and how this can have an impact on a variety of factors that influence the use of automated systems (See Table 5).

**Table 5:** ‘Modelling human–automation interaction’ cluster – summary of key research contributions

Article	Summary of work	Key themes	Level of automation
Hoff and Bashir (2015)	Systematic review and model creation of trust in automation, and how this interacts with reliance, system performance, and pre-existing knowledge	Modelling trust interactions, reliance	Unspecified
Parasuraman and Riley (1997)	Modelling trust, workload, risk, and the interactions between factors leading towards behavioural outcomes	Modelling trust interactions, reliance	Unspecified

Within this cluster, Parasuraman and Riley’s (1997) foundational paper outlined 14 current data-driven and hypothetical factors that interrelate to lead to reliance on an automated system. The authors outline how these factors come together to lead to potential decision biases that result in either an underreliance or overreliance on the automated system. The paper continues to be cited to refer to the varying factors that can influence reliance and highlight the need to optimise trust as a reflection of the capabilities of the automated system.

Eighteen years following this publication, Hoff and Bashir (2015) bring together the collective evidence on trust factors spanning the previous two decades. They expand on previous trust models by further defining the aspects of ‘system performance’ to include factors such as predictability, reliability, and validity, as well as outlining how pre-existing knowledge and design features can contribute towards trust and reliance. Both papers in this cluster provide cornerstones for the research community in a domain-agnostic fashion. Together, they provide foundational work for multiple disciplines to identify how they can design and measure interactions to optimise trust in their systems.

#### Risk

**Fig. 5:**
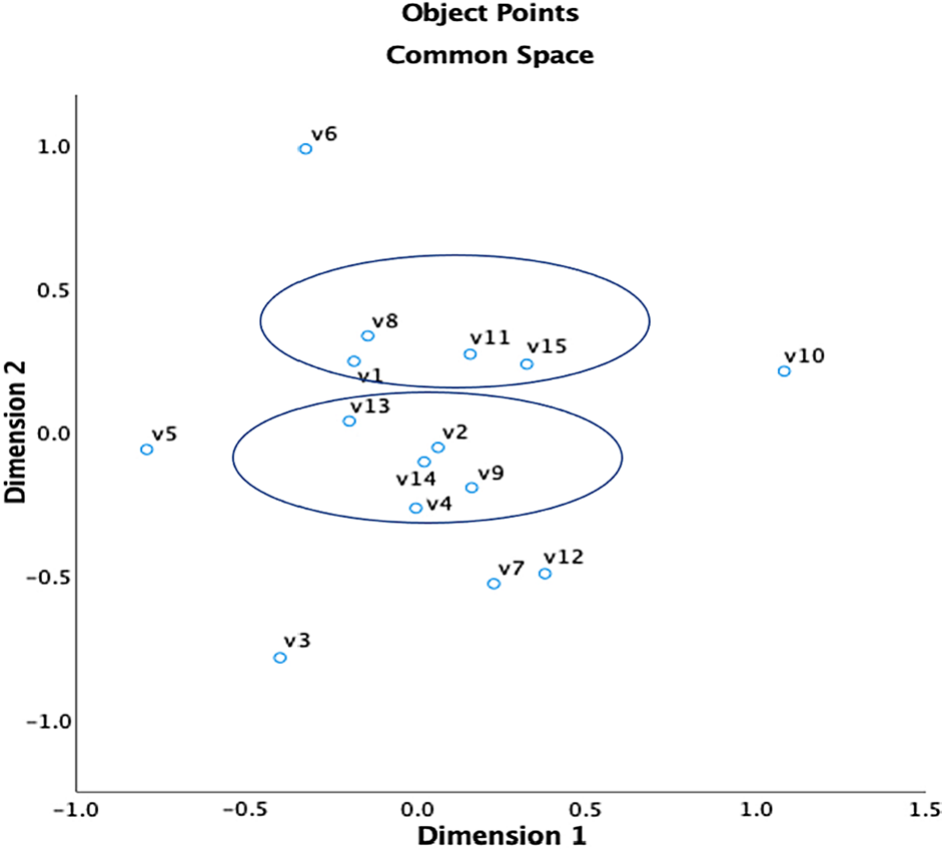
Risk: hierarchical clustering, Ward’s method. V1 = SAE (2019); V2 = Anderson et al. (2016); V3 = Bansal et al. (2016); V4 = Bonnefon et al. (2016); V5 = Choi and Ji (2015); V6 = Fagnant and Kockelman (2015); V7 = Haboucha et al. (2017); V8 = Hulse et al. (2018); V9 = Kalra and Paddock (2016); V10 = Kyriakidis et al. (2015); V11 = Lee and See (2004); V12 = Parasuraman and Riley (1997); V13 = Payre et al. (2014); V14 = Petit and Shladover (2015); V15 = Xu et al. (2018)

Amongst the most cited papers during the ‘risk’ keyword search, two clusters were identified from nine of these items (see Fig. 5). These clusters represent similarities across key papers within the domain and represent two major themes related to risk within AV research. The HCA clustering led to two clusters, namely, cluster 1—barriers, resilience, and regulation (V2, V4, V9, V13, V14); and cluster 2—user perceptions of AV capability (V1, V8, V11, 15).

##### Barriers, resilience, and regulation

The largest cluster within the risk search term relates to the demonstration that AVs are safe and effective in preventing additional or reducing current harm during their implementation and continued use (See Table 6).

**Table 6:** ‘Barriers, resilience and regulation’ cluster—summary of key research contributions

Article	Summary of work	Key themes	Level of automation
Anderson et al. (2014)	Outlining the promises and perils of AV technology, the law and legislative activity in the USA, the role of infrastructure and the issues of liability in AV operation	Liability, policy, law, infrastructure	2–5
Bonnefon et al. (2016)	Pinnacle paper addressing the public’s view of the social dilemma of AVs and the use of utilitarian algorithms to prevent NET loss of life	Ethics, algorithms, policy, safety	2–5
Kalra and Paddock (2016)	Assessing the frequency of miles required to demonstrate AV reliability	Reliability, safety	2–5
Payre et al. (2014)	Large-scale survey on attitudes and contributors towards the acceptance of fully autonomous vehicles. Investigates context, scenarios, and risk factors related to public uptake	Acceptance, usage, demographics	5
Petit and Shladover (2015)	Identifying potential vulnerabilities, feasibility of attacks, and associated consequences in a broad range of systems within AV operation	Security, safety, infrastructure	2–5

Bonnefon et al. (2016) and Payre et al. (2014) focus on the public views regarding the behaviour and functionality of AVs by investigating how AVs should behave when faced with situations related to the preservation of life, and the resultant public perceptions in a variety of scenarios and contexts that affect their intention to use AVs. Bonnefon et al. (2016) set the scene for ethical decision-making and providing a discourse around how AVs can be designed to reflect societies’ requirements for protecting drivers, pedestrians, or other road users during an unavoidable collision, whilst Payre et al. (2014) provide insight into what situations and contexts affect the public’s intention to buy and use AVs (e.g. congestion, highways, monotonous environments, and high frequency of hazards). Both articles outline how the public view AVs integrating into society and ensuring that they behave appropriately in a range of driving contexts.

To ensure that the autonomous vehicle (AV) technology meets public expectations and safety requirements, Kalra and Paddock (2016) and Petit and Shladover (2015) outline the challenges related to the resilience of AVs. Kalra and Paddock (2016) used mathematical modelling to estimate the test-driving mileage required to demonstrate the safe operation of AVs. They concluded that it would take a vast amount of time to demonstrate safety and emphasised on third-party testers to develop novel methods for testing vehicle safety. They continue by stating that uncertainty will persist and that the rollout of AV technology will incur risks if not correctly planned for. Petit and Shladover (2015) identified security risks targeting  13 potential vulnerabilities and outlined the likelihood, impact, and consequences of security failures in these systems. They provided the community with strategies for mitigating these threats, including hardware, software, and security measures. Both articles ensure that the physical systems of AVs meet acceptable standards. These articles provide a thorough insight into the issues that will arise in the years to come.

Finally, Anderson et al. (2014) investigated the legislation and liability issues surrounding AV deployment. They identify effects on crashes, mobility, traffic congestion, land use, energy and emissions, and overall costs. They evaluate what is currently supported by US law and provide a report on the issues that need to be tackled for AVs to protect stakeholders and the public. Their report provides additional qualitative data from stakeholders and formulates a set of recommendations for policymakers to address liability, insurance, and infrastructural matters on a state and national level. These papers collectively address the barriers to uptake from the perspectives of security, legality, public engagement, and reliability testing.

It is worth noting that in the context of AVs, the barriers to widespread adoption often stem from the complex interplay of AI with real-world scenarios. Challenges include addressing edge cases that AI models may find difficult to handle, ensuring robustness in unpredictable environments, and mitigating cybersecurity risks associated with AI-driven systems. Resilience, in this context, involves developing AI algorithms that can realistically handle unexpected situations, recover from faults, and continuously adapt to evolving road conditions, thereby bolstering the overall reliability of autonomous driving systems. Furthermore, the regulation of AVs is closely tied to the advancements and standards within the field of artificial intelligence. Governments and regulatory bodies are tasked with establishing frameworks that ensure the safety, ethical use, and responsible deployment of AI-powered AVs. Striking the right balance between innovation and risk mitigation is paramount, necessitating ongoing collaboration between the AI and regulatory communities to foster the development of a secure and accountable autonomous driving ecosystem.

##### User perceptions of AV capability

The second cluster for the risk search terms involves how users of autonomous vehicles perceive risk and how their own perceptions or risk taking align with the capability of the AV system (See Table 7). Both the SAE framework (SAE J3016B, 2018) and Lee and See (2004) provide a fundamental basis for outlining the relationship between a user and the AV system. Lee and See (2004) identify how users perceive the ability and performance of an autonomous system and outline the contributing factors that lead to better calibration of trust—*context, automation characteristics, and cognitive processes*. This has provided designers and manufacturers with a clear picture of how autonomous systems can communicate their intent and performance to ensure that a user correctly intervenes when the risk exceeds a certain threshold and does not intervene when not required to do so. The SAE framework (SAE J3016B 2018), in part, is an extension of this work, outlining the roles and responsibilities of both users and AVs in direct relation to AV functionality. The SAE framework outlines the levels of automation that are commonly used in current research discourse.

**Table 7:** ‘User perceptions of AV capability’ cluster—summary of key research contributions

Article	Summary of work	Key themes	Level of automation
SAE J3016B (2018)	Taxonomy and definitions for terms in autonomous vehicles. Levels of automation	Taxonomy, definitions	0–5
Hulse et al. (2018)	Perceptions of risk from a driver perspective, as well as road users such as passengers and pedestrians. Risk relationships with demographic characteristics and risk-taking behaviour	Context	5
Lee and See (2004)	Seminal piece on the calibration of trust onto reliance. Ensuring that trust is optimised for the context and reliability of the system	Context	Unspecified
Xu et al. (2018)	Identifying a model of behavioural intention to use self-driving vehicles including usefulness, ease of use, trust, and perceived safety	Context	3 & 5

Hulse et al. (2018) and Xu et al. (2018) provide a more user-centred evaluation of how perceptions of risk connect to intention-to-use AVs. Hulse et al. (2018) document the perceived risks of multiple road users including passengers and pedestrians and attempt to link this to demographical data such as gender, age, and risk propensity.

### Summary and application of clusters

Following a similar format to Parasuraman and Riley’s 1997 model of trust formation in automation (forming ‘prior to’ and ‘during’ interaction sections), the key themes found in the five clusters of this analysis are summarised in Fig. 5 and linked to intention to use.

During interaction, clusters often refer to the behaviours exhibited by the autonomous vehicle whilst in automated mode. This can refer to both the decisions the AV makes, and the actions that contribute to those actions (e.g. indicating and changing lane—action, decision—to overtake a vehicle (e.g. Körber et al. 2018; Muir and Moray 1996; Verberne et al. 2012; Waytz et al. 2014). This behaviour is mediated by human involvement, either through their awareness, or whether they can intervene at a given moment (e.g. Körber et al. 2018; Lee and Moray 1992; Muir and Moray 1996; Verberne et al. 2012). These factors are summarised by the human–AV interaction node and are directly influenced by regulatory features which inevitably define what features will be present within the vehicle, and the scenarios in which automation can be activated. How this interaction occurs and what boundaries are present will inevitably be in part influenced by local regulation and policy frameworks (Anderson et al. 2014).

**Fig. 6:**
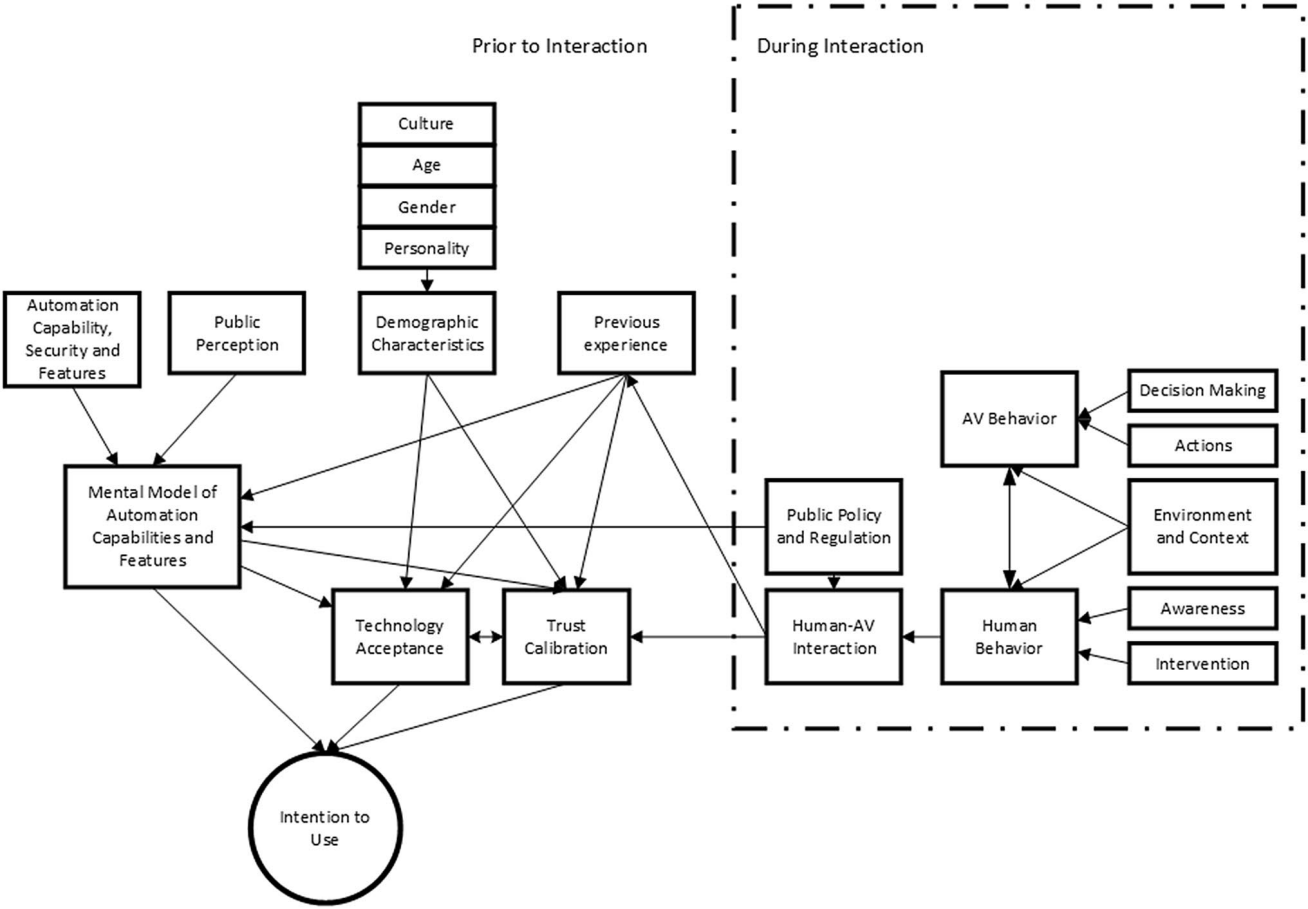
Key concepts explored in the realms of trust and risk of autonomous vehicles

Based on the identified literature, a mental model of capabilities is formed and regularly updated via previous experience, public perception, and the vehicles’ capabilities, security, and features (e.g. Bansal et al. 2016; Hoff and Bashir 2015; Kalra and Paddock 2016; Payre et al. 2014). These factors are identified as being key ingredients in trust formation (Hoff and Bashir 2015) and overall acceptance of the technology (Davis 1989), which in turn have been identified as influencing a user’s intention to use AVs (Xu et al. 2018). Demographic characteristics are also measured to be a contributory factor towards acceptance and trust calibration (e.g. Hulse et al. 2018, Lee and See 2004, Parasuraman and Riley 1997, Payre et al. 2014), included as gender, age, culture, and personality. Together, these clusters outline the key concepts explored in the realms of trust and risk of autonomous vehicles (see Fig. 6).

## Theoretical and practical implications

AVs are undergoing rapid development and are anticipated to yield benefits such as alleviating traffic congestion and reducing the incidence of road accidents (IIHS 2020). Our bibliometric analysis contributes to an inter-disciplinary understanding of the public perception of AVs, as well as future challenges pertaining to both societal and individual barriers to adoption.

To provide an interdisciplinary approach, we utilised the Web of Science database, focusing on trust and risk. We conducted a bibliometric and performance analysis to examine the conceptual and intellectual structures within autonomous vehicles across the following disciplines: engineering, social sciences, marketing, business, and infrastructure. By using Boolean operators, we have selected various keywords of Trust AND (Autonomous vehicle OR Automated Vehicle), Risk AND (Autonomous vehicle OR Automated Vehicle) to capture relevant studies.

As part of the bibliometric study search strategy and process, we examined the following components of the identified papers in the research domain: title, abstract, keywords and reference identifiers, and manuscripts. We selected studies that are (1) published in English and published in journals, conference proceedings, and reports. Indirect materials such as editorials or books were excluded from the analysis. To ensure the validity, two researchers collaborated on the inclusion and exclusion criteria as well as the selection of the studies. As a result, 936 studies were retrieved and utilised for performance analysis, with the most highly cited 30 papers being included in the co-citation analysis (Chabowski and Samiee 2020).

Trust and risk are two important determinants in users’ decision-making processes regarding emerging technologies such as AI (e.g. Shin 2021) and blockchain (e.g. Shin and Bianco 2020; Shin and Hwang 2020). Through bibliometric analysis and synthesis of the literature, our analysis offers an overview of understanding of trust and risk perceptions within autonomous vehicles. Additionally, a deeper understanding of public perception in relation to autonomous vehicles is attained, encompassing its historical conception and development. Moreover, our research facilitates the proposal of a comprehensive model of public perception in autonomous vehicles outlining the key themes discovered in the five clusters (see Sect. 3.3 and Fig. 6 for a summary of key topics).

### Theoretical implications

By reviewing the literature under the ‘trust’ search category, the research made an important theoretical contribution by informing understanding on the key factors impacting trust in AVs. Our study showed a significant role of *AV behavioural aspects* in shaping trust, which has been emphasised theoretically and empirically in our reviewed papers. It does so by integrating the role of human drivers in automated technology and how drivers’ behaviour interacts with perceived trust (Körber et al. 2018; Verberne et al. 2012). Further, *predicting the uptake of AVs* was a primary theme in our analysis. The literature identified key factors that affect public uptake and acceptance, such as the role of the driving environment and the demographics of the users (Bansal et al. 2016; Payre et al. 2014). Finally, modelling human–automation trust appeared as the main cluster in the ‘trust’ category (Hoff and Bashir 2015; Parasuraman and Riley 1997). Top cited papers in this category were reviewed to provide a road map outlining how to design systems and measure interactions to optimise trust.

Our analysis revealed two main clusters under the ‘risk’ category: *barriers, resilience and regulation* and *user perceptions of AV capability*. Although the risk category was a well-established concept in our reviewed papers, these contributions have always limited their definition of risk perception at an individual level (Bonnefon et al. 2016; Petit and Shladover 2015). Risk attitudes have a social structure so that, oftentimes, even technologies that are relatively safe (from the technical point of view) can induce strong public concern. This phenomenon has been discussed as a social amplification of the risk (Kasperson et al. 1988, 2022). When the risk is socially amplified, its perception becomes emotional rather than analytical; thus, the AV research community may need to further examine whether social amplification exists in AV and propose potential methods and approaches to mitigate it.

The theoretical implications of our results extend beyond the empirical findings, shedding light on fundamental aspects of trust and risk in the context of AVs. Our results confirm current findings which emphasise the significant role of AV behavioural aspects in shaping trust, contributing to theories surrounding human–automation interaction (Lee et al. 2021). This underscores the dynamic interplay between technology and human users, highlighting the need for a nuanced understanding of how drivers’ behaviour influences trust perceptions. The integration of human drivers into the theoretical framework of automated technology challenges traditional notions of trust solely rooted in technical reliability, paving the way for a more holistic model that considers the symbiotic relationship between humans and AVs.

Furthermore, our exploration of risk perception introduces a theoretical dimension by highlighting the social structure of risk attitudes. The acknowledgment that even safe technologies can evoke strong public concerns aligns with theories of risk communication and social amplification of risk (Lundgren and McMakin 2018). This insight challenges conventional views that risk perception is solely determined by technical factors, emphasising the need for a comprehensive theoretical framework that incorporates the social dynamics influencing how risks are perceived and communicated in the context of AVs. In this way, our study provides a theoretical foundation for future research on trust and risk in the evolving landscape of autonomous driving technology.

### Practical implications

The incoming mass adoption of artificial intelligence in all the areas of vehicle automation has also important implications for how trust can be affected. For instance, the explainability of autonomous driving operations will be critical to constructing a trust relation between humans and AVs (Naiseh et al. 2020; Shin et al. 2022a, b). Thus, future contributions in this field might need to address trust issues that arise from human–explanation interaction, and do not expect drivers or passengers to be passive operators able to discern the operational intricacies of the AV. For instance, explainable intelligent assistant interfaces may adopt friction design principles to nudge operators towards AVs’ explanation to calibrate trust and avoid overlooking or missing critical information.

Furthermore, risk perception of passengers and drivers in the context of AVs holds practical implications for the design and deployment of these technologies. Clear communication of safety features is paramount, ensuring that AVs possess easily understandable systems that inform users about emergency protocols, fail-safe mechanisms, and the vehicle’s capability to handle various scenarios (Deb et al. 2020). Implementing real-time risk feedback mechanisms can enhance user understanding of the AV’s decision-making process, providing immediate explanations for safety–critical decisions and deviations from normal driving behaviour (Nguyen et al. 2023). Additionally, designing interfaces that facilitate collaboration between humans and AVs can positively impact risk perception by allowing passengers to interact with the system, make informed decisions, and intervene when necessary (Shin 2023). Gradual exposure and familiarisation strategies, coupled with training and education programmes, can help users acclimate to AV technology, building trust and lowering perceived risks over time (Naiseh and Shukla 2023; Shin 2020). User-friendly safety alerts and public awareness campaigns that openly address concerns contribute to a more informed and accepting user base, while establishing regulatory standards for risk communication ensures consistency and reliability across different autonomous systems (Wang et al. 2020).

## Future research directions

It has been shown that new products and services, such as AVs face resistance in terms of public acceptance due to their inherent risk (Naiseh and Shukla 2023). This inherent risk is generally regarded as being a composite of several categories of risks. Kaplan et al. (1974) identified five types of risk perception: *performance, physical, financial, psychological, and social*. Researchers have confirmed that risk perception and its intensity can generally be placed in one or more of these categories. To the best of our knowledge, to date, no empirical studies have applied risk categories while examining AV driver behavioural intentions. Such studies may provide deeper knowledge about understanding AV customers’ attitudes and behaviours.

In one related study, user-centric design principles have been shown to be effective in guiding the development of algorithms, ensuring that interfaces effectively communicate the intentions and actions of autonomous systems, fostering transparency and user trust (Shin et al. 2022a, b). However, future research on AVs may also need to prioritize understanding and enhancing the adoption and acceptance processes of the algorithms governing these vehicles, placing a strong emphasis on the influence of public awareness. For instance, consider a scenario where an AV encounters a complex traffic situation requiring it to make a nuanced decision, such as yielding the right of way in an ambiguous intersection. Future research could focus on developing algorithms that not only make the optimal decision in such scenarios, but also effectively communicate the rationale behind the decision to passengers and pedestrians. This user-centric approach would contribute to building trust and confidence in the capabilities of autonomous systems, ultimately influencing the widespread acceptance of AVs.

Another interesting direction for future research is to delve into the dynamics of human–AI interaction (e.g. Faber and Lierop 2020), particularly focusing on understanding how passengers and pedestrians engage with AVs. This research could explore a multitude of aspects, including the communication interfaces between AVs and pedestrians, as well as the emotional and psychological responses of passengers during AV journeys. Initiatives to raise public awareness about the capabilities and limitations of autonomous algorithms are also critical. Educational programs and campaigns can contribute to dispelling misconceptions, building trust, and creating an informed public perception of AI in AVs. Additionally, ethical considerations in algorithmic decision-making for AVs must be addressed, aligning algorithms with societal values to enhance public acceptance.

Finally, our proposed model (Fig. 6) provides a foundation for future empirical testing through user experiments and structural equation modelling. For instance, our model underscores the significance of personal and cultural differences in trust calibration. Subsequent research endeavours could delve into cultural and personal variances in trust calibration using AV algorithms, examining how cultural factors can contribute to higher or lower levels of trust. This exploration has the potential to inform the development of international regulatory frameworks for the deployment and operation of AI algorithms in AVs, thereby contributing to the establishment of a cohesive and safe global autonomous driving ecosystem.

## Study limitations

Like any other study, this study has some limitations. First of all, to extract the studies, we used the Web of Science database. Using different databases such as Cochrane Library or Scopus might yield researchers to obtain different results. Second, we have performed our bibliometric, and performance analysis to identify conceptual and intellectual structures of trust and risk narratives from a context/country-specific approach. Having a country or context-based bibliometric study might expand the findings of our study by providing further comparative results. Similarly, one of our inclusion/exclusion criteria was to include only English studies: including studies in different languages might equally be important and help researchers to identify new paths for future studies within the AV domain.

## Conclusion

In this study, hierarchical cluster analysis was selected to conduct the bibliometric analysis. Bibliometric studies, no matter what analysis method is utilised, are highly dependent on the most highly cited articles that are included in the analysis; therefore, the changes in the sample sizes might change the results. Therefore, we encourage future researchers to conduct different forms of bibliometric studies, such as multi-dimensional scaling (MDS) or combining both MDS and HCA to have a two-mode network evaluation.

## Data Availability

Not applicable.

## Footnotes

1 Web of Science offers a wide collection of articles, conference proceedings, books, and patents (over 34,888) in social sciences, engineering, arts, and humanities and the most comprehensive coverage of natural sciences and engineering, computer science, materials sciences, patents, and data sets.

2 To gather the critical keywords, the selection of the database, and the core categories that have been selected, the authors have consulted experts (academics and practitioners in the field) within the AV research field between March and April 2021.
